# Hotels re-explored: Experience and influence of reciprocity and social normative appeals

**DOI:** 10.1371/journal.pone.0289602

**Published:** 2023-12-07

**Authors:** Malin Ekelund, Magnus Bergquist

**Affiliations:** Department of Psychology, University of Gothenburg, Gothenburg, Sweden; UCL: University College London, UNITED KINGDOM

## Abstract

In this paper we report two high-powered and pre-registered experiments, testing the robustness and conceptual development of reciprocity and social norm appeals. Both experiments assessed both psychological processes for complying with these appeals and pro-environmental behavioral intention in tourism settings. In Experiment 1 (N = 2004), participants reported lower psychological reactance levels after learning that the hotel engaged in resource conservation (i.e., indirect homeomorphic reciprocity). No statistically significant effect was obtained for either obligatory motivation, prosocial motivation, skepticism, or behavioral intentions to reuse hotel towels. Importantly, high baseline intention of reusing hotel towels might have limited the effect of appeals. Therefore, we targeted meat consumption in Experiment 2 (n = 2540). Results first showed stronger obligatory and prosocial motivation for all three reciprocity appeals, compared to the standard appeal. No statistically significant results were found for either reactance or skepticism. Finally, after learning that the hotel had made a financial contribution to an environmental organization (i.e., indirect heteromorphic reciprocity) participants showed reduced meat consumption intentions compared to the standard appeal. Overall, the results provide initial evidence for conceptually refining the norm of reciprocity to encourage pro-environmental behaviors and for understanding the underlying psychological processes.

## Introduction

Human activity has indisputably caused climate change [1]. A sector that is rapidly growing, thus accelerating global carbon emissions, is the tourism sector, which is currently estimated to be responsible for 8–11% of global emissions of greenhouse gases [2, 3]. By way of illustration, one hotel night is equal to 28–57 kg CO_2_ per occupied room. Motivating pro-environmental behavioral change at the individual level is a central component to help mitigate further emissions [4].

Past research has developed and assessed numerous intervention strategies aimed at making behavior more sustainable [e.g., 5–8]. These interventions have targeted behaviors such as energy and water conservation [e.g., 9, 10], food waste reduction [e.g., 11, 12], promoting ethical food [e.g., 13], and reducing meat consumption [e.g., 14]. To motivate such pro-environmental behavioral change, various interventions, often described as “nudging”, such as implementing smaller plate sizes [e.g., 11], and asking hotel guests to opt out of automatic everyday cleaning has been successful [e.g., 15]. The overall effectiveness of the so-called “nudging” techniques has however been severely criticized. Studies report that results are gravely decreased when these interventions are implemented on a large scale [16] and that meta-analytic evidence is nullified after adjusting for publication bias [17, 18]. One potential explanation for these inconsistent effects is that nudging refers to a plethora of social influence techniques with are context dependent and rely on different psychological motives [19, 20]. In aiming to assess such techniques, a recent second-order meta-analysis reported that social norm-based interventions have great potential in encouraging pro-environmental behaviors [5]. Yet, social normative influence has shown to be dispersive [7, 21]. Past research shows that the influential power of social norms depend on if the norm promotes or prevents a behavior [21], if the norm is inducing a common goal or not [22], and if the norm is static or dynamic [23]. Hence, an adequate understanding of the processes motivating people to (not) follow social norms is central for crafting social norms-based interventions that actually changes behaviors. In this paper, we seek to assess such underlying motives, both for the descriptive social norm, but also for the norm of reciprocity. Given the number of studies on norms and pro-environmental behaviors, it is somewhat surprising that few studies have focused on the norm of reciprocity. In essence, the norm of reciprocity builds on people’s motivation to reciprocate a favor. Past research has implemented reciprocity norms with persuasive messages based on incentives and cooperation to motivate pro-environmental behaviors [24, 25]. The norm of reciprocity has great potential to encourage behavioral change, the effect is noteworthy and has been shown to be robust in replications [24, 26–29]. However, the norm of reciprocity has gained far less interest than social norms and there is a lack of studies evaluating different types of reciprocity.

In this article, we report two pre-registered and highly powered experiments, replicating and extending past research, testing the robustness and processes of social norms-based persuasive to promote pro-environmental behaviors. Specifically, we conceptually replicated Goldstein et al. [9, 30] by utilizing social norms and the norm of reciprocity, while extending the previous research with two new reciprocity appeals. Further extension to the research was done by testing the experience of the appeals through measurements of motivation and resistance. In Experiment 2, we further extended past research by targeting intended decreased meat consumption.

### Descriptive social norms

Social norms refer to the unwritten rules and expectations of a group or society, as they are currently being practiced [31]. These norms are often informal and may not be explicitly stated, but they still exert a powerful influence on individuals’ behavior. Descriptive social norms refer to what other people typically do, and people are motivated to conform to them by a desire to adapt to a situation. Knowing that many others perform a behavior serves as a cue about adaptive or effective behaviors in that situation [32, 33]. As such, when people follow a descriptive norm, they are taking a decisional shortcut and choosing an option that is likely to be effective in the given situation [32]. For example, setting the hotel room air condition temperature at 20°C after being informed that most guests find that temperature to be the most comfortable [34]. When a behavior is desirable, but not what most people do—as is often the case with pro-environmental behaviors—the use of a descriptive dynamic norm may be fruitful. Dynamic norms highlight that a norm is changing in a specific direction and that more and more individuals are engaging in the desired behavior [23]. For instance, a dynamic norm intervention for sustainable consumption in a café influenced consumers to avoid disposable cups and opt for reusable alternatives [35].

In the context of sustainable resource consumption, past research using descriptive norm interventions has tested behaviors such as the adoption of reusable take-away boxes [36], the reduction of air conditioning temperatures [34], asking for a “doggy-bag” [37], and the selection of vegetarian food [14]. For research applied to the tourism sector, towel reuse emerges as the behavior that has by far gained the most interest [6]. One of the earliest and most cited articles on towel reuse, and the precursor to the present research, is Goldstein et al. [9]. In this work, Goldstein and colleagues examined appeals requesting hotel guests to participate in an environmental conservation program by reusing their towels. Results showed that the normative appeal of a specific-descriptive norm message (i.e., a provincial norm stating that 75% of guest who stayed in the same hotel room reused their towels) yielded the highest towel reuse (49.3%) compared to an industry standard message (i.e., no normative information, which yielded 37.2%).

Replicational attempts of Goldstein et al. [9] findings show both consistent [e.g., 38–40] and conflicting results [41] of the provincial norm effect. In synthesizing this line of research, a Bayesian meta-analysis confirmed that social descriptive norms are indeed more effective overall than standard environmental appeals [42]. These results have, however, been criticized for the assumptions regarding the Bayesian synthesis used and the possibility of publication bias [43], thus, rendering the results inconclusive. In corroborating these finding, Nisa et al. [44] reported a small, yet statistically significant, meta-analytic overall effect of descriptive social norms (*d* = -0.25).

### The norm of reciprocity

Reciprocity is a universal social rule building on a sense of social obligation where people should return favors [45–47]. Reciprocity has been theorized to be the fundamental part of human nature [48], and has been shown to take place even when the favor-giver will not find out whether the favor is reciprocated [49]. The norm of reciprocity supervises relationships between individuals as well as relationships between individuals and businesses [30].

Reciprocity often plays a role in people agreeing to requests. In particular, requesters can use the norm to their advantage by doing a small, unexpected favor for the person they are asking [19]. The Door-in-the-Face technique utilizes this mechanism by first asking for a large objectionable request, and when denied, doing the “favor” of asking for a smaller, more reasonable, request; increasing the likelihood that people will comply with the small request [26, 27, see also 50]. A typical reciprocal exchange entails that Party A provides Party B with a favor, making Party B feel indebted to Party A, creating an obligation on Party B to return the favor to Party A [24]. Obligation (i.e., personal norms) have shown to be a substantial and proximal predictor of various pro-environmental behaviors [51, 52], and more specifically, mediate the intention to reuse hotel towels [53]. Nevertheless, few studies within environmental psychology that we know of have induced obligations via reciprocity [25]. Also, there are many different types of reciprocal exchanges [54].

For the present research we focus on *what* is given and to *whom*. Equivalence in reciprocity is defined as the extent to which what is exchanged is directly comparable to what was received. It ranges from homeomorphic to heteromorphic [45], where homeomorphic reciprocity refers to when the action of exchange is concretely alike or identical (“tat-for-tat”), and heteromorphic refers to when the action is concretely different but with the same value (“tit-for-tat”). Direct reciprocity can be broadly defined as a mechanism where individuals help those who have previously helped them. Indirect reciprocity can be described as the tendency for individuals to help others who have previously helped someone else [55, 56].

In the context of sustainable tourism, reciprocity has been tested as an intervention strategy for behaviors such as vegetarian food choice [25], and contribution to a national park [57]. In Goldstein et al. [30] the reciprocity message (i.e., informing guests that on behalf of the guests and themselves, the hotel has donated to an environmental organization and wants help to redeem the cost) yielded the second-highest towel reuse (45.2%) compared to what they called an “environmental cooperation” message (30.7%), i.e., a statement that the hotel will donate on behalf of the guests *after*, rather than *before*, which is the principle of reciprocity.

Given the attention in both game theory [e.g., 58] and social influence [46, 50], it is noteworthy that only a few studies have tested the norm of reciprocity as an intervention technique for promoting pro-environmental behaviors. In this article, we advance the current state of knowledge by examining the persuasive impact of different types of norms of reciprocity.

## Overview of studies

In two experiments (see Table 1. for a summary of hypotheses), we examined the replicability, generalizability, and effectiveness of descriptive social norms and reciprocity norms for behavior interventions in the context of sustainable tourism. Furthermore, we wanted to evaluate the *experience* of norm interventions by assessing plausible psychological processes associated with being exposed to these interventions, hence, we extended past research and conducted online experiments, contrasting the field experiments of Goldstein et al. [9, 30].

**Table 1:** Summary of hypotheses and explorative analyses.

Hypotheses and explorative analyses	Prediction
**Experiment 1**	
H1: Descriptive provincial norm appeal	Higher towel reuse intention than the standard appeal
H2: Reciprocation appeal (indirect heteromorphic/financial contribution)	Higher towel reuse intention than the standard appeal
H3: Modified reciprocal appeals (indirect homeomorphic/resource conservation and direct homeomorphic/made it easy)	Different intention to reuse hotel towels compared to the standard appeal
E1: Descriptive provincial norm and reciprocity appeals	Differences in intention to reuse hotel towels
E2: Ratings of 1) obligatory motivation, 2) pro-social motivation, 3) psychological reactance*, and 4) skepticism	Differences between the five appeals
E3: Moderation by participants’ environmental self-identity	Impact on intention to reuse hotel towels across appeals
**Experiment 2**	
H1: Descriptive provincial norm appeal	More vegetarian elections than the standard appeal
H2.1: Reciprocation appeal (indirect homeomorphic/meat reduction)	More vegetarian elections than the standard appeal
H2.2: Reciprocation appeal (indirect heteromorphic/financial contribution)*	More vegetarian elections than the standard appeal
H2.3: Reciprocation appeal (direct homeomorphic/made it easy)	More vegetarian elections than the standard appeal
E1: Descriptive provincial norm and reciprocity appeals	Differences in vegetarian elections
E2: Ratings of 1) obligatory motivation*, 2) pro-social motivation*, 3) psychological reactance*, and 4) skepticism	Differences between the five appeals

*Note*.

* Indicates statistically significant results.

In Experiment 1, we sought to conceptually replicate the effectiveness of the three appeals used by Goldstein et al. [9, 30]: 1) “standard environmental message”, 2) “provincial descriptive norm”, and 3) “the norm of reciprocity” on participants intention to reuse their hotel towels. Second, we sought to extend Goldstein et al. [9, 30] by also testing two modified versions of the reciprocity appeal. Third, and specific for the present research, we sought to investigate participants’ experience of the appeals.

In Experiment 2, we sought to further extend and replicate Experiment 1 with a behavior that is more topical and personal than hotel towel reuse, namely choosing a vegetarian option at the hotel breakfast.

### The modified reciprocity appeals

Goldstein’s et al. [24, 30] reciprocity-by-proxy message could be conceptually described as inducing the norm of indirect heteromorphic reciprocity by stating that the hotel has *financially* contributed to an *environmental organization* on the behalf of the guests and the hotel *in advance*. Hence, the guests are expected to reciprocate to the hotel by reusing their towels because the hotel has made a financial contribution to a third party. We are testing this message exactly as Goldstein et al. [30] for Experiment 1, and slightly modified to fit the topic of Experiment 2.

Building on research suggesting that non-monetary gifts are more effective in stimulating effort than monetary gifts [e.g., 59, 60], our first modified reciprocity message is conceptualized indirect homeomorphic reciprocity. We developed a modified appeal where the financial incentive is replaced by a non-financial incentive. More specifically, our message stated that the hotel has *decreased* the energy and water consumption for the sake of the environment by having a towel reuse program (Experiment 1) or less meat options on the menu (Experiment 2) and is asking the guests to reuse their towels/choose a vegetarian option to help them *further decrease* it. Hence, the guests are expected to reciprocate to the hotel (and the environment) because of the effort of the hotel to help save the environment.

Based on past research indicating a stronger effect for direct compared to indirect reciprocity [47, 50, 61], our second reciprocity message is utilizing direct homeomorphic reciprocity. We modified this appeal to incorporate a situation where the hotel first helps the guest, and then, the guest is asked to reciprocate to the hotel. More specifically, our message stated that the hotel is committed to preserving the environment and has therefore *in advance* made it *easy* for the participant to join them in the effort *simply* by reusing their towels (Experiment 1) or choosing a vegetarian option (Experiment 2) to help them decrease their energy and water consumption/carbon footprint. Hence, the guests are expected to reciprocate to the hotel because the hotel is making it easy for the guests to help save the environment.

### The experience of the appeals—Motivation and resistance

To explore how people experience the descriptive norm and reciprocity appeals, we draw upon motivations from the Self-Determination Theory (SDT; [62]). SDT explains how the social environment nurtures or prevents individuals’ motivation and subsequently effects emotions, cognitions, and behaviors. The theory emphasizes a fundamental distinction between two forms of motivation—autonomous and controlled—that explain why individuals differ in experiences and self-regulatory processes and what results from them. Specifically, autonomous motivation arises when individuals have the sense of the action being voluntary and something they *want* to do, whereas controlled motivation arises when individuals have the sense of the action being obligatory and something they *have* to do [63, 64]. It is important to identify the type of motivation that guides prosocial behavior, as different types of motivation can lead to different outcomes [64]. For the present context, will the appeals make individuals feel motivated to reuse their hotel towels or choose a vegetarian option because they *want to* and/or because the feel like they *should*? For example, will the indirect heteromorphic reciprocal appeal induce more of a sense of obligation than the descriptive norm appeal, due to the underlying mechanism of indebtedness of the reciprocity norm—and vice versa, will the descriptive norm appeal induce more of a sense of prosocial, voluntary, motivation than the reciprocity appeal, due to the underlying nature of a motivation to conform to what other people do.

Persuasive attempts may backfire due to psychological reactance [65]. Any message trying to convince an individual to engage in a desired behavior may arouse a motivation in the individual to reject it—or even increase the unwanted behavior in a boomerang effect [e.g., 66–68]. Psychological reactance theory [65, 69, 70] asserts that individuals perceive threats to their freedom when they are being persuaded to change their attitudes or behaviors in a certain way. In the present context, all the appeals are trying to convince the participants to take a desired course of action to “help save the environment”. Research evaluating the impact of psychological reactance produced by normative appeals on intentions to act pro-environmentally showed that descriptive appeals elicited less reactance than injunctive appeals [71]. Further resistance to persuasion may be posed by skepticism. Hotels applying towel reuse programs, and other sustainable initiatives, have been accused of “greenwashing” (i.e., deceptive use of marketing or PR to create a false impression of being environmentally friendly). Being skeptical of hotels’ green initiatives, such as believing they have a financial ulterior motive, has shown to lead to decreased intention to participate in a towel reuse program as well as intention to revisit the hotel [72].

## Experiment 1

In experiment 1, we first conceptually tried to replicate the effectiveness of the three appeals used by Goldstein et al. [9, 30]: 1) “standard environmental message”, 2) “provincial descriptive norm”, and 3) “the norm of reciprocity” on participants intention to reuse their hotel towels. Second, we wanted to extend Goldstein et al. [9, 30] by also testing two modified versions of the reciprocity appeal. Third, we wanted to investigate participants’ experience of the appeals.

The following hypotheses and exploratory questions were tested:

Hypothesis 1: The descriptive provincial norm appeal [9] will yield higher towel reuse intention than the standard appeal.
Hypothesis 2: The reciprocation appeal [30] of indirect heteromorphic (i.e., financial contribution) will yield higher towel reuse intention than the standard appeal.
Hypothesis 3: We expect that the intention to reuse hotel towels will differ between either of the two modified reciprocal appeals (indirect homeomorphic/resource conservation and direct homeomorphic/made it easy) and the standard appeal.
Explorative analysis 1: We will explore if the descriptive provincial norm and the three reciprocity appeals differ in intention to reuse hotel towels.
Explorative analysis 2: We will explore if ratings of 1) obligatory motivation, 2) pro-social motivation, 3) psychological reactance, and 4) skepticism differ between the five appeals.
Explorative analysis 3: We will explore if the intention to reuse hotel towels across appeals will be moderated by participants environmental self-identity.

The hypotheses and data-analysis plan were pre-registered and can be found at https://osf.io/s3jyr/?view_only=4ef5a619694141ae922158742f4a1fb7.

## Method

### Participants and design

We based our power analysis on the weakest effect of interest in Goldstein et al. [9] (i.e., the difference between the standard environmental appeal and the descriptive provincial normative appeal, Cohen’s *d* = .27. Odds ratio: calculated as (493/507)/(372/628) = 1.64 transforms to d = .27. After a Bonferroni adjusted alpha value (α/5), we used G*Power to calculate the number of participants needed to obtain the estimated effect size in pair-wise post-hoc tests (tails = 2, d = .27, α = .01, β-1 = .8, allocation ratio = 1/1). Results showed that we needed 323 participants in each condition, adding up to a total of 1615 participants. To further increase power, whilst staying within the budget limitations for the present project, we recruited 2000 participants. The final sample consisted of 2004 participants living in the United States. Participants were recruited from Prolific (www.prolific.co) and were compensated with £0.77 for completing the survey, which on average took 3.21 minutes to complete. Participants included 903 women, 1061 men, 11 transgender, 21 who identified as “other”, and 8 persons who preferred not to specify their gender. The average age of participants was 39.7 years (*SD* = 13.3, range: 18–91).

### Materials and procedure

The experiment was conducted via a survey that was administered online using Qualtrics. Each participant was randomly assigned to one of the five appeals. The main sentiment of the recruitment text that was presented to participants on Prolific, and on the first page of the Qualtrics survey, was: “We are conducting a study about staying at a hotel”. After reading the introduction text in Qualtrics the participants proceeded to give their informed consent to partake in the study and read the introductory text:

Imagine that you are staying at a mid-range hotel for three nights on your own.
On the next page you will see a video, please imagine that it is you in the video. In the video you are walking into your hotel room for the first time and as you check out the bathroom you see a message.
After watching the video, you will be asked to answer some questions regarding that message and the hotel. At the end of the survey there will be a few questions about yourself.

#### The appeals: Video and text

We used short videos depicting the appeals on a sign hanging in a hotel bathroom to increase realism. Participants watched one of the five videos after reading the introductory text. The message appeals were: a) a standard (control) message (same as Goldstein et al. [9]), b) a descriptive provincial norm message (same as Goldstein et al. [9]), c) an indirect heteromorphic reciprocity message (same as Goldstein et al. [30]), d) an indirect homeomorphic reciprocity message (created for this study), and e) a direct homeomorphic reciprocity message (created for this study). The message that the participants saw on the sign in the video was displayed to them again, in text, above every question so that they could fully read the text before answering the questions. The videos can be found at https://osf.io/s3jyr/?view_only=4ef5a619694141ae922158742f4a1fb7.

Standard message:

HELP SAVE THE ENVIRONMENT.

You can show your respect for nature and help save the environment by reusing your towels during your stay.

Descriptive provincial norm message:

JOIN YOUR FELLOW GUESTS IN HELPING TO SAVE THE ENVIRONMENT.

Almost 75% of the guests who stayed in this room (#214) participated in our new resource savings program by using their towels more than once. You can join your fellow guests in this program to help save the environment by reusing your towels during your stay.

Reciprocity (indirect heteromorphic/financial contribution) message:

WE’RE DOING OUR PART FOR THE ENVIRONMENT. CAN WE COUNT ON YOU?

Because we are committed to preserving the environment, we have made a financial contribution to a nonprofit environmental protection organization on behalf of the hotel and its guests. If you would like to help us in recovering the expense, while conserving natural resources, please reuse your towels during your stay.

Reciprocity (indirect homeomorphic/resource conservation) message:

WE’RE DOING OUR PART FOR THE ENVIRONMENT. CAN WE COUNT ON YOU?

Because we are committed to preserving the environment, we have decreased our energy and water consumption with our towel reuse program. If you would like to help us continue this decrease, while conserving natural resources, please reuse your towels during your stay.

Reciprocity (direct homeomorphic/made it easy) message:

WE’VE MADE IT EASY FOR YOU TO HELP SAVE THE ENVIRONMENT.

Because we are committed to preserving the environment, we have made it easy for you to join us in this effort. Simply reuse your towels during your stay and you will help us to decrease our energy and water consumption.

The back of each sign had the same text as Goldstein et al. [9]:

DID YOU KNOW that if most of this hotel’s guests participate in our resource savings program, it would save the environment 72,000 gallons of water and 39 barrels of oil, and would prevent nearly 480 gallons of detergent from being released into the environment this year alone?

And the same prompt on how to reuse or not reuse the towels (adapted for the present study):

- A towel hanging up means "I will use again".
- A towel on the floor means "I’d like a new one."

The prompt was also displayed to the participants, together with the message, before each question.

#### Motivation

The items that followed the video were obligatory motivation and prosocial motivation (randomized order). The items for obligatory motivation were adapted from Eisenberger et al. [73]. Specifically, participants rated how the message made them feel (1 = *strongly disagree*, 7 = *strongly agree*) on the statements: (a) I feel obliged to reuse my towels, (b) I owe it to the hotel to reuse my towels, (c) I owe it to nature to reuse my towels, and (d) I feel guilty if I do not reuse my towels. The items evaluating participants prosocial motivation were adapted from Grant [74]. Specifically, participants rated how the message made them feel (1 = *strongly disagree*, 7 = *strongly agree*) on the statements: (a) I care about benefiting others by reusing my towels, (b) I want to help others by reusing my towels, (c) I want to have positive impact on others by reusing my towels, and (d) It is important to me to do good for others by reusing my towels. The mean of these ratings formed two composite measures of obligatory motivation (Cronbach’s α = .84), and prosocial motivation (α = .98).

#### Dependent variable: Reuse intention

The participants then answered two items regarding their intention to reuse their hotel towels. Specifically, 1) “How would you handle your towel when staying at this hotel?” (1 = *definitely get new towels*, 7 = *definitely reuse towels*). 2) “How many of your towels would you reuse?” (0 = *none of the towels*, 6 = *all 5 towels)*. The mean of these two ratings formed an index variable of reuse intention (α = .75).

#### Resistance

The items that followed reuse intention were psychological reactance and skepticism (randomized order). The items evaluating psychological reactance were adapted from Dillard and Shen [75]. Specifically, participants indicated to what extent they agreed (1 = *strongly disagree*, 7 = *strongly agree*) with the statements: (a) The message threatened my freedom to choose, (b) The message tried to make a decision for me, (c) The message tried to manipulate me, and (d) The message tried to pressure me. The items evaluating skepticism were adapted from Rahman et al. [72], which in turn was adapted from Mohr et al. [76]. Specifically, participants indicated to what extent they agreed (1 = *strongly disagree*, 7 = *strongly agree*) with the statements: (a) The hotel’s claim for its environmental concern is true (reverse coded), (b) The hotel’s claim for its environmental concern is intended to mislead, (c) The hotel’s claim for its environmental concern is exaggerated, and (d) I do not believe that the hotel truly cares about the environment as it claims. The mean of these ratings formed two composite measures of psychological reactance (α = .86), and skepticism (α = .75).

#### Demographics

Participants then answered five demographic items: age, gender, education, average annual nights in hotels, and environmental self-identity [77]. Specifically, participants rated their environmental self-identity (1 = *strongly disagree*, 7 = *strongly agree*) on the statements: (a) Acting environmentally friendly is an important part of who I am, (b) I am the type of person who acts environmentally friendly, and (c) I see myself as an environmentally friendly person. The mean of these ratings created a composite measure of environmental self-identity (α = .95).

## Results and discussion

All confirmatory analyses followed the preregistered analysis plan. Data is available at https://osf.io/s3jyr/?view_only=4ef5a619694141ae922158742f4a1fb7.

### Hypothesis 1–3 and explorative analysis 1

**Fig 1:**
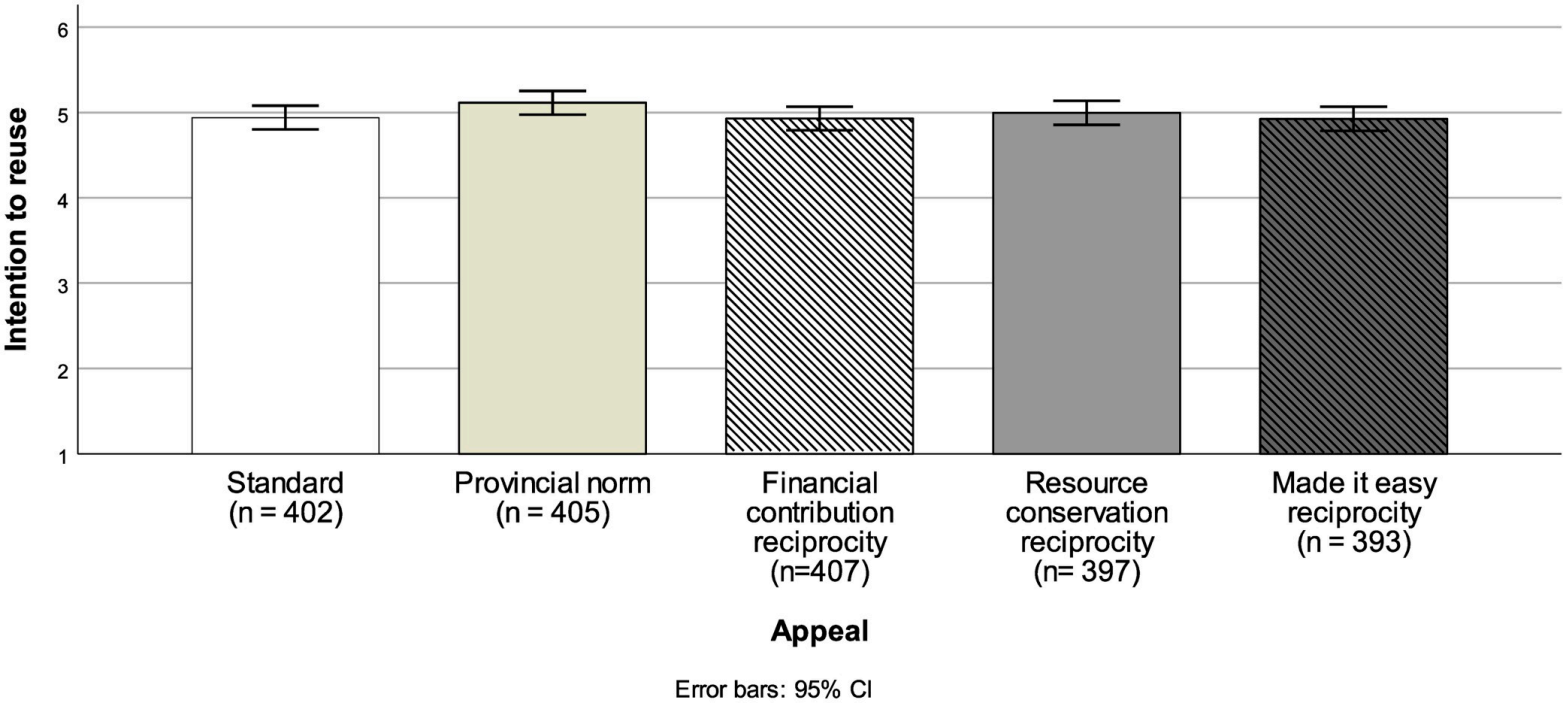
Intention to reuse as a function of the five appeals. *Note*. Error bars represent 95% confidence intervals.

To test hypotheses 1–3 and explorative analysis 1, we ran an independent one-way ANOVA with five conditions (Appeal: standard vs. provincial norm vs. financial contribution reciprocity vs. resource conservation reciprocity vs. made it easy reciprocity) for ratings on reuse intention. Results revealed no significant difference across the conditions (*F*(4, 1999) = 1.25, *p* = .29, *ƞ*_*p*_^2^ = .002). Hence, we obtained no statistically significant difference, nor any noticeable descriptive effects, between the five appeals on participants intentions to reuse their hotel towels (see Fig 1). Consequently, we did not replicate the finding of Goldstein et al. [9, 30] that a descriptive provincial norm, and an indirect heteromorphic reciprocity norm (i.e., financial contribution) yielded higher towel reuse than the standard appeal. Likewise, our two modified reciprocity appeals did not yield higher reuse intention than the standard appeal.

One important difference between this study and past studies on message appeals for hotel towel re-usage is that we assessed intentions rather than actual behavior. Importantly, a recent meta-analysis including 572 studies on pro-environmental behavior reported that descriptive norms are more strongly related to intentions than behaviors [51]. Hence, stronger rather than weaker effects should be expected in this study compared to past studies [42]. Another important difference between this and previous studies is that re-use intentions were higher in both the control and experimental conditions (about 75%) compared to the average re-use behavior reported by Scheibehenne et al. [42], 50% in the control condition and 56% in the social norms condition. Related to this, past research shows that social norms are less influential for subjects holding strong personal norms, as they might already engage in the targeted behavior [4, 78]. Therefore, in Experiment 2, we are testing a behavior that we expect to have a lower baseline than towel reuse, namely asking meat-eating participants to choose a vegetarian option at the hotel breakfast.

### Explorative analysis 2

For the second explorative analysis, we ran an independent one-way MANOVA testing if the five appeals differ on ratings of 1) obligatory motivation, 2) prosocial motivation, 3) skepticism, and 4) reactance, see Table 2 for mean ratings. Results showed non-significant results for obligatory motivation (*F*(4, 1999) = 0.06, *p* = .99, *ƞ*_*p*_^2^ < .001), prosocial motivation (*F*(4, 1999) = 0.17, *p* = .96, *ƞ*_*p*_^2^ < .001), and skepticism (*F*(4, 1999) = 1.29, *p* = .30, *ƞ*_*p*_^2^ = .002). For reactance, we obtained a statistically significant effect (*F*(4, 1999) = 3.73, *p* = .005, *ƞ*_*p*_^2^ = .007). A follow-up Tukey HSD test indicated that the only statistically significant post-hoc effect was lower reactance in the resource conservation reciprocity appeal (*M* = 2.59, *SD* = 0.71) compared to the standard appeal (*M* = 2.89, *SD* = 0.70, *d* = 0.43, 95% CI [0.29, 0.57], *p* = .023). That is, the resource conservation appeal elicited lower psychological reactance than the standard appeal. A possible explanation may be that the standard message used the phrasing “you can show your respect for nature”, which implies that if you do not follow the appeal, you are not showing respect. The message further does not politely ask the guest to reuse their towels, like the other messages that end with a “please reuse your towels”. Taken together, asking participants using forceful language, and without asking politely invokes stronger reactance than when less forceful and more polite language is used. This is in line with research drawing upon politeness theory [79, 80] as an explanation of the process of reactance to persuasion attempts [81]. When messages with more forceful and controlling language is used, compared to less forceful messages, more reactance is felt [81].

**Table 2:** Mean ratings of obligatory motivation, prosocial motivation, psychological reactance, and skepticism as a function of message appeal.

Message Measure	Standard Message (*n* = 402)	Descriptive Norm (*n* = 405)	Financial contribution Reciprocity (*n* = 407)	Resource conservation Reciprocity (*n* = 397)	Made it easy reciprocity (*n* = 393)
Obligatory motivation	4.62 (1.53)	4.59 (1.46)	4.58 (1.50)	4.59 (1.48)	4.57 (1.49)
Prosocial motivation	5.39 (1.49)	5.46 (1.50)	5.44 (1.41)	5.43 (1.54)	5.45 (1.45)
Reactance	2.89 (1.41)	2.81 (1.46)	2.86 (1.45)	2.59* (1.33)	2.77 (1.42)
Skepticism	3.45 (1.25)	3.31 (1.27)	3.36 (1.28)	3.32 (1.27)	3.27 (1.20)

*Note*. Standard deviations appear in parentheses next to means.

* Indicates statistical significance.

### Exploratory analysis 3

For Exploratory analysis 3, a moderation analysis, using Hayes’ [82] PROCESS macro (Model 1; 5,000 bootstrap samples), was run to test a model with message appeal as independent variable, reuse intention as dependent variable, and environmental self-identity as a moderator of the effect of message appeal on reuse intention. There was no significant interaction between message appeal and participants’ environmental self-identity on reuse intention, *ΔR*^*2*^ = .000, *F*(1, 2000) = 0.85 *p* = .356. Hence, participants’ environmental self-identity did not affect their intention to reuse towels as a function of message appeal. It has been theorized that individuals with a strong environmental self-identity will more strongly see themselves as the type of person who will act environmentally friendly and consequently be more likely to act pro-environmental [77]. In the present research we were interested to see if the effect of message appeal on reuse intention was moderated by participants’ environmental self-identity. We did not see an effect in the present research and will therefore not use the concept of environmental self-identity in study 2.

## Experiment 2

Experiment 2 attempted to replicate Experiment 1 with another pro-environmental behavior—asking meat-eating participants to choose a vegetarian option at the hotel breakfast. We expected this behavior to have a lower baseline than towel reuse. This is crucial as behaviors with lower baselines imply more variance for improvement.

The following hypotheses and exploratory questions were tested:

Hypothesis 1: The descriptive dynamic norm appeal will yield more vegetarian elections than the standard appeal.
Hypothesis 2.1: The indirect homeomorphic (i.e., resource conservation) reciprocity appeal will yield more vegetarian elections than the standard appeal.
Hypothesis 2.2: The indirect heteromorphic (i.e., financial contribution) reciprocity appeal will yield more vegetarian elections than the standard appeal.
Hypothesis 2.3: The direct homeomorphic (i.e., made it easy) reciprocity appeal will yield more vegetarian elections than the standard appeal.
Explorative analysis 1: We will explore if the descriptive dynamic norm appeal and the three reciprocity appeals differ in vegetarian elections.
Explorative analysis 2: We will explore if ratings of 1) obligatory motivation, 2) pro-social motivation, 3) psychological reactance, and 4) skepticism differ between the five appeals.

The hypotheses and data-analysis plan were pre-registered and can be found at https://osf.io/s3jyr/?view_only=4ef5a619694141ae922158742f4a1fb7.

## Method

### Participants and design

We based the power calculation on a second-order meta-analysis of environmental behavior that found an overall effect of *d* = .18 [5]. Results showed that we needed 489 participants in each condition, 2445 in total. We recruited 3000 participants to further increase power and to account for the exclusion of vegetarians, vegans, pescatarians, and failed attention check (see Exclusion). There was a lag on Prolific which added another 9 participants. Due to the exclusion of 475 non-meat-eating participants and 227 meat-eaters who failed the attention check, the final sample was below the 2445 calculated for power. Hence, we recruited 301 additional participants, of which we excluded 48 non-meat-eaters and 20 meat-eaters who failed the attention check.

The final sample comprised of 2540 participants living in the UK. Participants were recruited from Prolific and were compensated with £0.77 for completing the survey, which on average took 3.42 minutes to complete. Participants included 1339 women, 1177 men, 8 transgender, 9 who identified as “other”, and 7 persons who preferred not to specify their gender. The average age of participants was 42.35 years (*SD* = 13.6, range: 18–87).

### Materials and procedure

The experiment was conducted via a survey that was administered online using Qualtrics. Each participant was randomly assigned to one of the five appeals. The main sentiment of the recruitment text that was presented to participants on Prolific, and on the first page of the Qualtrics survey, was: “We are conducting a study about staying at a hotel”. After reading the introduction text in Qualtrics the participants proceeded to give their informed consent to partake in the study and read the introductory text:

Imagine that you are staying at a mid-range hotel for three nights on your own.
On the next page you will see a video, please imagine that it is you in the video. In the video you are sitting down in the hotel restaurant to order your breakfast, on the first page of the menu you see a message. After watching the video, you will be asked to answer some questions regarding that message and the hotel. At the end of the survey there will be a few questions about yourself.

#### The appeals: Video and text

As in Experiment 1, we used short videos depicting the appeals to increase realism. This time the appeals were written on the first page of a breakfast menu. Participants watched one of the five videos after reading the introductory text.

The message that was on the menu in the video was displayed to the participants again, above every question, so that they could fully read the text before answering the questions. The videos can be found at https://osf.io/s3jyr/?view_only=4ef5a619694141ae922158742f4a1fb7.

Standard message:

HELP SAVE THE ENVIRONMENT.

You can show your respect for nature and help save the environment by choosing a vegetarian option.

Descriptive dynamic norm message:

JOIN YOUR FELLOW GUESTS IN HELPING TO SAVE THE ENVIRONMENT.

More and more guests choose a vegetarian option for breakfast. You can join your fellow guests and help conserve the environment by choosing a vegetarian option.

Reciprocity (indirect heteromorphic/financial contribution) message:

WE’RE DOING OUR PART FOR THE ENVIRONMENT. CAN WE COUNT ON YOU?

Because we are committed to preserving the environment, we have made a financial contribution to a nonprofit environmental protection organization on behalf of the hotel and its guests. If you would like to help us in recovering the expense, while conserving natural resources, please choose a vegetarian option.

Reciprocity (indirect homeomorphic/meat reduction) message:

WE’RE DOING OUR PART FOR THE ENVIRONMENT. CAN WE COUNT ON YOU?

Because we are committed to preserving the environment, we have reduced the meat options on our menu. If you would like to help us further reduce our carbon footprint, while conserving natural resources, please choose a vegetarian option.

Reciprocity (direct homeomorphic/made it easy) message:

WE’VE MADE IT EASY FOR YOU TO HELP SAVE THE ENVIRONMENT.

Because we are committed to preserving the environment, we have made it easy for you to help us in this effort by having many vegetarian dishes on our menu. Simply choose a vegetarian option and you will help us reduce our carbon footprint.

#### Motivation

The items that followed the video were obligatory motivation and prosocial motivation (randomized order), the same as Experiment 1 except that “reusing towels” were switched to “choosing a vegetarian option”. The mean of these ratings formed two composite measures of obligatory motivation (Cronbach’s α = .88), and prosocial motivation (α = .98).

#### Dependent variable: Vegetarian choice

The participants chose between four dishes, two meat options (Hotel Inn Grill, Eggs Benedict) and two vegetarian variants of the meat options (Hotel Inn Grill vegetarian, Eggs Florentine). The items formed a dichotomous variable of vegetarian/meat.

#### Resistance

The items that followed reuse intention were psychological reactance and skepticism (randomized order), exactly the same as Experiment 1. The mean of these ratings formed two composite measures of psychological reactance (α = .91), and skepticism (α = .79).

#### Attention check

Participants were asked to identify which message (out of the 5) they had seen in the video and before the questions.

#### Demographics

Participants answered four demographic items: age, gender, education, and what food group they identified with (vegetarian, vegan or 100% plant-based, pescetarian, meat-eater, other (which they were asked to specify)).

## Results and discussion

### Hypothesis 1

A 2 × 2 contingency Chi-square test was conducted to determine if the descriptive dynamic appeal yielded more vegetarian elections than the standard appeal. Results indicated that there was not a significant difference between the two appeals, *χ*^*2*^ (1, *N* = 1035) = 0.3, *p* = .57, *d* = 0.04, 95% CI [-0.08, 0.16]. Hence, Hypothesis 1 was not supported.

### Hypothesis 2.1

A 2 × 2 contingency Chi-square test was conducted to determine if the meat reduction reciprocity appeal yielded more vegetarian elections than the standard appeal. Results indicated that there was not a significant difference between the two appeals, *χ*^*2*^ (1, *N =* 1013) = 0.6, *p =* .43, *d* = 0.05, 95% CI [-0.07, 0.17]. Hence, Hypothesis 2.1 was not supported.

### Hypothesis 2.2

A 2 × 2 contingency Chi-square test was conducted to determine if the financial contribution reciprocity appeal yielded more vegetarian elections than the standard appeal. Results indicated that there was a significant difference between the two appeals, *χ*^*2*^ (1, *N =* 985) = 7.6, *p =* .006, *d* = 0.18, 95% CI [0.05, 0.30], as we obtained a p-value lower than the adjusted criterium (α/n_test_ = .0125). That is, the indirect heteromorphic appeal was more effective than the standard appeal to persuade meat-eaters to choose a vegetarian option over a meat option. Hence, Hypothesis 2.2 was supported.

### Hypothesis 2.3

A 2 × 2 contingency Chi-square test was conducted to determine if the made it easy reciprocity appeal yielded more vegetarian elections than the standard appeal. Results indicated that there was not a significant difference between the two appeals, *χ*^*2*^ (1, *N =* 1033) = 2.5, *p =* .17, *d* = 0.09, 95% CI [-0.04, 0.21]. Hence, Hypothesis 2.3 was not supported.

In summarizing the four confirmatory hypotheses, the only message appeal that significantly yielded more vegetarian elections than the standard appeal was the indirect heteromorphic (i.e., financial contribution) reciprocity appeal. Giving a donation to an environmental organization on behalf of the hotel and the guests in advance elicited more cooperation, compared to a standard appeal. This is partly in line with the results of Goldstein et al. [30], their reciprocity appeal yielded the second most towel reuse compared to the standard message, and the descriptive norm message yielded the most. Whereas the descriptive norm message in the present study did not significantly yield more vegetarian elections than the standard message. In Goldstein et al. [30] the results are for towel reuse, in the present experiment we tested vegetarian food elections. Our results are further in line with a field study by Lange et al. [25], applying indirect heteromorphic reciprocity to promote vegetarian food options. The authors found that donating to an environmental organization in advance increased vegetarian elections by 50% compared to a control condition [25].

### Exploratory analysis 1

A 4 × 2 contingency Chi-square test was conducted to determine if the descriptive dynamic norm appeal and the three reciprocity appeals differed in vegetarian elections. There was a significant difference *χ*^*2*^ (3, *N =* 2032) = 11.9, *p =* .008. However, when conducting the post hoc test, whilst controlling for type 1 error inflation with an adjusted *p*-value of .006, the differences between the appeals were not statistically significant, all *p’s* = > .006.

### Exploratory analysis 2

A one-way MANOVA was conducted comparing ratings of 1) obligatory motivation, 2) pro-social motivation, 3) psychological reactance, and 4) skepticism between the five message appeals (see Table 3 for means in all conditions). There was a significant effect of message appeal *F* (16, 2535) = 3.88, *p* = < .001, *η*^*2*^_*p*_ = 0.00. Test of between-subjects effects showed that the results was significant for obligatory motivation *F* (4, 2535) = 9.40, *p* = < .001, *η*^*2*^_*p*_ = 0.01, 90% CI [0.01, 0.02), for pro-social motivation *F* (4, 2535) = 5.53, *p* = < .001, *η*^*2*^_*p*_ = 0.00, 90% CI [0.00, 0.01], and for psychological reactance *F* (4, 2535) = 3.02, *p* = .02, *η*^*2*^_*p*_ = 0.00, 90% CI [0.00, 0.01]. For skepticism, the effect was non-significant, *p* = .19. A follow-up Tukey HSD test indicated that for obligatory motivation, the standard appeal (*M* = 3.10, *SD* = 1.50) was significantly lower than the financial contribution reciprocity appeal (*M* = 3.49, *SD* = 1.53), *p* = < .001, *d* = -0.26, 95% CI [-0.38, -0.13], the meat reduction reciprocity appeal (*M* = 3.45, *SD* = 1.52), *p* = .002, *d* = -0.23, 95% CI [-0.36, -0.11], and the made it easy reciprocity appeal (*M* = 3.60, *SD* = 1.56), *p* = < .001, *d* = -0.33 95% CI [-0.45, -0.20]. That is, the standard appeal elicited lower obligatory motivation than all the reciprocity appeals, but not for the descriptive dynamic norm appeal (*M* = 3.23, *SD* = 1.45, *p* = .66, *d* = 0.09, 95% CI [-0.03, 0.21]. The descriptive dynamic norm appeal elicited lower obligatory motivation than the financial contribution reciprocity appeal, *p* = .040, *d* = -0.17, 95% CI [-0.30, -0.05], and the made it easy reciprocity appeal, *p* = < .001, *d* = -0.15, 95% CI [-0.27, -0.03]. There were no differences between the reciprocity appeals (all *p’s* > .05).

**Table 3:** Mean ratings of obligatory motivation, prosocial motivation, psychological reactance, and skepticism as a function of message appeal.

Message Measure	Standard Message (*n* = 508)	Descriptive Norm (*n* = 527)	Financial contribution Reciprocity (*n* = 477)	Meat reduction Reciprocity (*n* = 503)	Made it easy reciprocity (*n* = 525)
Obligatory motivation	3.10 (1.50)	3.23 (1.45)	3.49* (1.53)	3.45* (1.52)	3.60* (1.56)
Prosocial motivation	3.42 (1.62)	3.51 (1.60)	3.76* (1.68)	3.60 (1.60)	3.82* (1.64)
Reactance	4.62 (1.50)	4.55 (1.52)	4.71 (1.51)	4.64 (1.40)	4.40 (1.55)
Skepticism	3.68 (1.15)	3.68 (1.12)	3.63 (1.17)	3.54 (1.10)	3.57 (1.11)

*Note*. Standard deviations appear in parentheses next to means.

* Indicates statistical significance.

In sum, the standard appeal elicited the lowest obligatory motivation, and the made it easy reciprocity appeal elicited the highest. That the reciprocity appeals induced higher obligatory motivation is in line with the theory of reciprocity and the underlying mechanism of indebtedness [45, 83]. Further, all the reciprocity appeals induced obligatory motivation to a comparable degree, indicating no difference between the different types of reciprocity.

For pro-social motivation, the made it easy reciprocity appeal (*M* = 3.82, *SD* = 1.64) elicited the highest mean score, which was statistically significantly higher than the standard appeal (*M* = 3.42, *SD* = 1.62), *p* = < .001, *d* = 0.25, 95% CI [0.12, 0.37], and the descriptive dynamic norm (*M* = 3.51, *SD* = 1.60), *p* = .016, *d* = 0.19, 95% CI [0.07, 0.31]. The financial contribution reciprocity appeal (*M* = 3.76, *SD* = 1.68) significantly elicited higher pro-social motivation than the standard appeal, *p* = .008, *d* = 0.21, 95% CI [0.08, 0.33]. The meat reduction reciprocity appeal (*M* = 3.60, *SD* = 1.60) did not statistically differ from the other appeals.

In sum, the made it easy reciprocity appeal stimulated the highest pro-social motivation, whilst the standard appeal stimulated the lowest. Descriptively, all the reciprocity appeals elicited higher pro-social motivation than the standard appeal and the descriptive dynamic norm appeal. It has been shown that reciprocity not only induces a sense of indebtedness, but that it also induces a sense of cooperation and prosocial motivation [24, 84]. The mean scores for prosocial motivation were higher than for obligatory motivation on all reciprocity appeals, indicating that the appeals more strongly evoked a sense of prosocial motivation than obligatory motivation.

For psychological reactance, the meat reduction reciprocity appeal (*M* = 4.40, *SD* = 1.55) elicited the lowest reactance, which was statistically significantly lower than the financial contribution reciprocity appeal (*M* = 4.71, *SD* = 1.51), *p* = .011, *d* = -0.20, 95% CI [-0.33, -0.08], which had the highest ratings. There were no other differences between the appeals and ratings of psychological reactance (all *p’s* > .05).

## General discussion

Building on past research, reporting that social norm-based interventions have the potentiality to change behaviors [e.g., 7, 85], we set out to conceptually replicate and extend the studies by Goldstein et al. [9, 30]. In two pre-registered and highly powered experiments, we tested both how people experience and are influenced by message appeals utilizing social norms and norms of reciprocity. The targeted behaviors where intentions to reuse hotel towels and intentions to choose vegetarian food. As a baseline appeal we used an “industry standard” appeal, simply asking people to reuse their towels or choose vegetarian food for the sake of the environment. This appeal was compared to a descriptive provincial norm (Experiment 1) and a descriptive dynamic norm (Experiment 2), and three versions of the reciprocity norm (i.e., indirect heteromorphic/financial contribution, indirect homeomorphic/resource consternation or meat reduction, and direct homeomorphic/made it easy).

In Experiment 1, neither the social norm appeal nor the reciprocity appeals were more persuasive than a standard appeal to influence participants intention to reuse their towels. Hence, we did not replicate the effect of Goldstein et al. [9, 30]. The explorative analysis of our extension to past research found that resource conservation reciprocity appeals (i.e., indirect homeomorphic reciprocity) elicited lower psychological reactance than the standard appeal. One potential explanation for the null effect in intention is the relatively high baseline of hotel towel reuse intentions (M = 5 on a 7-point scale). We therefore conducted Experiment 2, targeting reduced meat consumption, which we suspected would have a lower baseline, and hence more variance for strengthened intentions. In Experiment 2, the financial contribution reciprocity appeal was more persuasive, and consequently yielded more vegetarian elections, than the standard appeal. Hence, partially replicating the effect of Goldstein et al. [30] with a different behavior. Moreover, explorative analyses first showed that people felt stronger obligatory motivation after being exposed to either of the reciprocity appeals compared to the standard appeal. Second, pro-social motivations were stronger after being exposed to either the financial contribution or the made it easy reciprocity appeal compared to the standard appeal. Finally, neither the social norm appeal nor the reciprocity appeals differed from the standard appeal in psychological reactance and skepticism toward the sender of the appeal.

Descriptively, comparing the mean ratings of obligatory motivation, prosocial motivation, and psychological reactance in relations to the appeals between Experiment 1 and Experiment 2 shows an interesting pattern (see Tables 2 and 3 for comparisons). In Experiment 1, with UK citizens and towel reuse, the ratings for both obligatory and prosocial motivation across appeals were high, whilst ratings for reactance were low. In Experiment 2, with US (meat-eating) citizens and vegetarian selection, the opposite was true, ratings for reactance were high, whilst ratings for both obligatory and prosocial motivation were low. A possible explanation for why motivation is higher for hotel towel reuse than meat-reduction may be warm glow, a positive anticipated “feel good” for helping the environment [86]. Research has shown that warm glow is associated with pro-environmental behaviors that are of low-cost (i.e., switching off lights), but not of high-cost (i.e., purchase green energy), arguably, meat-reduction for meat-eaters is a high-cost behavior when compared with towel-reuse. Further, there is research showing that eating meat leads people to withdraw their moral concern [87], and that morality is the lowest of motivations [88], given the lower means of obligatory motivations (with moral concerns in the questions, e.g., “I would feel guilty if I do not choose a vegetarian option”) this could further be a reason for the difference in results in the present research. An interpretation of why the opposite pattern emerges regarding reactance, is that for the arguably more person relevant behavior of opting for a meatless breakfast than reusing a towel evokes more reactance for the reason of it being just that, of personal importance [65, 69]. Additionally, a hotel message on towel reuse is common practice, whilst asking meat-eaters to choose a vegetarian option is not. The participants in Experiment 1 have likely been exposed to similar messages numerous times, hence, the mere exposure effect [89] could have had a positive impact on felt psychological reactance [90]. For participants in Experiment 2, it is more likely that it was the first time seeing such a message. Noteworthy for Experiment 2, the financial contribution appeal had the highest ratings of psychological reactance whilst it, paradoxically, also was the only appeal that elicited more vegetarian elections than the standard appeal. In general, reactance is a barrier for a persuasive message, making people perceive it as a threat to their freedom of choice and consequently lead to less intention to act according to the message [75, 91] or even a boomerang effect, where the unwanted behavior is performed to a higher degree [e.g., 68]. One possible explanation for this seemingly contradictory finding comes from the process explanation of reciprocity. The persuasive power of reciprocity comes from the motivation to “give back” or to “reestablish social balance”, after receiving a favor. The received favor might be perceived as threatening one’s freedom, as it implies social indebtedness. Importantly, people are given the opportunity to reestablish social balance by reciprocating. This makes the reciprocity technique different from other social influence techniques, where reactance is expected to limit social influence, and consequently worth to further explore for interventions targeting pro-environmental behaviors.

Another important difference between the experiments is the baseline of pro-environmental behavioral intentions. The overall vegetarian choice, across conditions, was 38,3% compared to the overall reuse intention of 75% in Experiment 1. High baselines imply that there is less variance for strengthening intentions, which might partly explain the lack of effect in Experiment 1, while we obtained an effect in Experiment 2. Further, the psychological barriers, factors that prevent a different or new behavior to occur [92], to meat consumption reduction are feasibly stronger than for towel reuse. An example of a strong psychological barrier is habit, a recent review of the research regarding psychological barriers to meat reduction found that habit is the strongest psychological barrier to change, and that values and attitudes potentially acts as moderating variables [93]. In the seven studies included in the review, framing and messaging was the most frequently recommended intervention. However, none of the messaging in the studies centered around reciprocity, and to the best of our knowledge there is only one study, prior to ours, utilizing reciprocity targeting meat reduction [25]. The process of reciprocity, specifically indebtedness and the motivation to give back, may be a way to overcome habits for behaviors in tourist settings. Habits for pro-environmental behaviors in general is an underexplored research area [94] and needs to be directly studied.

### Limitations and future research

The present research studied the effect on intention of behavior (Experiment 1) and choice (Experiment 2) in online experiments. Whilst it gave the opportunity to extend past research and explore participants’ experience of the different appeals, it did not measure actual behavior as is done in field experiments. Research has, however, shown that social norms interventions have equal—or even stronger—effects on intentions than they do on actual behavior [e.g., 51, 78]. Therefore, measuring intention rather than behavior is an unsatisfactory explanation for the null results.

Given the lower ecological validity of an online experiment compared to a field experiment, the present research used short videos as stimulus to increase the realism. However, further research evaluating intentions and actual behavior, especially for the reciprocity appeals, is needed. For instance, reciprocity norms could be examined for a variety of pro-environmental behaviors in both field experiments and online experiments. Different types of wording and framing of the messages should also be examined. For example, in the messages in the present research, based on Goldstein et al. [9], the framing is to “preserve the environment” and “help save the environment”. This is very broad and unspecific; it would be of value to explore a more specific framing in the messages. For instance, “help conserve water for the sake of [the present city]”. Further, the beneficiary in the financial contribution reciprocity message could be specified, however, it needs to be an organization that is appealing to most people, otherwise it could backfire [24].

The heteromorphic reciprocity appeals were operationalized as monetary incentives, while the homeomorphic reciprocity appeals were non-monetary, introducing a confounder in the effect. We cannot establish if the effects we saw from the reciprocity appeals were due to the monetary incentive or homogeneity/heterogeneity in terms of resources. Future studies should test different types of reciprocity without monetary incentives or include monetary incentives in all of them.

In Experiment 1, environmental self-identity did not moderate the intentions to reuse towels, and therefore we did not use the concept in Experiment 2. In hindsight, it would have been of value to test for the potential moderating effect of environmental self-identity on vegetarian election, as meat-reduction for meat-eaters is plausibly more influenced by personal norms than towel-reuse for a non-specific segment of the population. Hence, for future studies within the present domain, we recommend evaluating participants’ personal norms to see if they moderate their intention or behavior, as reported by past research [e.g., 53].

### Industry implications

Our findings suggest that for more personal relevant behaviors (i.e., meat reduction), utilizing heteromorphic indirect reciprocity, that is, a financial contribution by the establishment to an environmental organization on behalf of its guests is a fruitful way to encourage guests to choose a meat-free option. Whilst the financial contribution reciprocity appeal yielded the most vegetarian selections it also elicited the most psychological reactance. Using reciprocity could be a potential buffer against reactance in relation to persuasive messages. Whilst our initial findings regarding such a buffer are intriguing, they should be interpreted cautiously and serve as a starting point for further investigation. To establish a more comprehensive understanding of the potential buffering effect of reciprocity on psychological reactance for persuasive messages, future studies should include non-meat eaters and employ confirmatory hypotheses to validate the results.

It has become more common to ask hotel guests to opt in for cleaning than to by default clean rooms. This in turn leads to more towel reuse. Research has shown that a “green default” (i.e., information with an environmental appeal), compared to a default without an environmental appeal, reduced the percentage of cleans to 32% of room cleans being requested [15]. To add the norm of reciprocity to such green defaults may be a good way to further increase participation.

## Conclusions

We aimed to conceptually replicate and extend Goldstein et al. [9, 30] by conducting two high-powered preregistered experiments, where we tested the robustness and experience of persuasive appeals in promoting pro-environmental behaviors. First, we found that appeals based on social and reciprocity norms had no statistically significant effect on intentions to re-use hotel towels, in Experiment 1. Second, in Experiment 2, which targeted reduced meat consumption during a hotel stay, results showed that, compared to the standard appeal, participants’ intended meat consumption decreased after the hotel reported making a financial contribution to an environmental organization. In assessing the motivational process following these appeals, we found that participants reported stronger obligatory motivation and pro-social motives after being exposed to the reciprocity appeals, but not the social norm appeal. Overall, this research provides a more conservative estimate of the potential effect of using explicit appeals as a means to stimulate pro-environmental behaviors in tourism settings. Exploratory findings pave way for further exploration of a potential buffering effect of financial contribution reciprocity on psychological reactance for high-cost pro-environmental behaviors.
